# Strain-controlled superconductivity in epitaxially grown thin films of 1*T*-TaS_2_

**DOI:** 10.1038/s41598-025-19901-y

**Published:** 2025-10-15

**Authors:** Yelyzaveta Chernolevska, Anže Mraz, Rok Venturini, Bojan Ambrožič, Tomaž Mertelj, Goran Dražič, Damjan Svetin, Damjan Vengust, Hsin-Chia Ho, Matjaž Spreitzer, Dragan Mihailovic

**Affiliations:** 1 Department of Complex Matter, Jozef Stefan Institute, Jamova 39, 1000 Ljubljana, Slovenia; 2 Faculty for Electrical Engineering, University of Ljubljana, Tržaška 25, 1000 Ljubljana, Slovenia; 3 Faculty for Mathematics and Physics, University of Ljubljana, Jadranska 19, 1000 Ljubljana, Slovenia; 4 CENN Nanocenter, Jamova 39, 1000 Ljubljana, Slovenia; 5 Department of Materials Chemistry, National Institute of Chemistry, 1001 Ljubljana, Slovenia; 6 Advanced Materials Department, Jozef Stefan Institute, Jamova 39, 1000 Ljubljana, Slovenia

**Keywords:** Electronic properties and materials, Surfaces, interfaces and thin films, Superconducting properties and materials

## Abstract

1*T*-TaS_2_ is a prototype layered material with a rich phase diagram that includes multiple charge density wave (CDW) transitions and technologically important metastable states. It also supports a superconducting phase induced by hydrostatic pressure, cation substitution, intercalation, or doping. Thin 1*T*-TaS_2_ crystals deposited on various substrates exhibit transition temperatures that are strongly dependent on the substrate-induced strain, and depart from bulk transition temperatures in a way that is not clearly understood at present. Here we show that thin polycrystalline films of 1*T*-TaS_2_ grown by molecular beam epitaxy on (LaAlO_3_)_0.3_(Sr_2_TaAlO_6_)_0.7_ (LSAT) substrates have a suppressed CDW transition to a commensurate phase. Instead, resistivity, magnetoresistance, and critical current measurements reveal metallic behavior with an onset to a superconducting state below $\:{T}_{c}=3.8$ K. The appearance of superconductivity is suggested to be driven by the *in-plane tensile* differential strain exerted on the 1*T*-TaS_2_ film by the LSAT substrate during cooling, which in turn results in a strongly amplified *out-of-plane compressive* strain triggered by the Poisson effect, combined with traceable signs of intercalation with La and Sr atoms from the substrate. The experiments suggest that *tensile* substrate strain may be usefully applied for achieving desirable Functional properties that are otherwise accessible through hydrostatic pressure, and generally for investigating of the effects of anisotropic strain in 2D materials and monolayer stacks or heterostructures.

**Supplementary Information:**

The online version contains supplementary material available at 10.1038/s41598-025-19901-y.

## Introduction

**Fig. 1:**
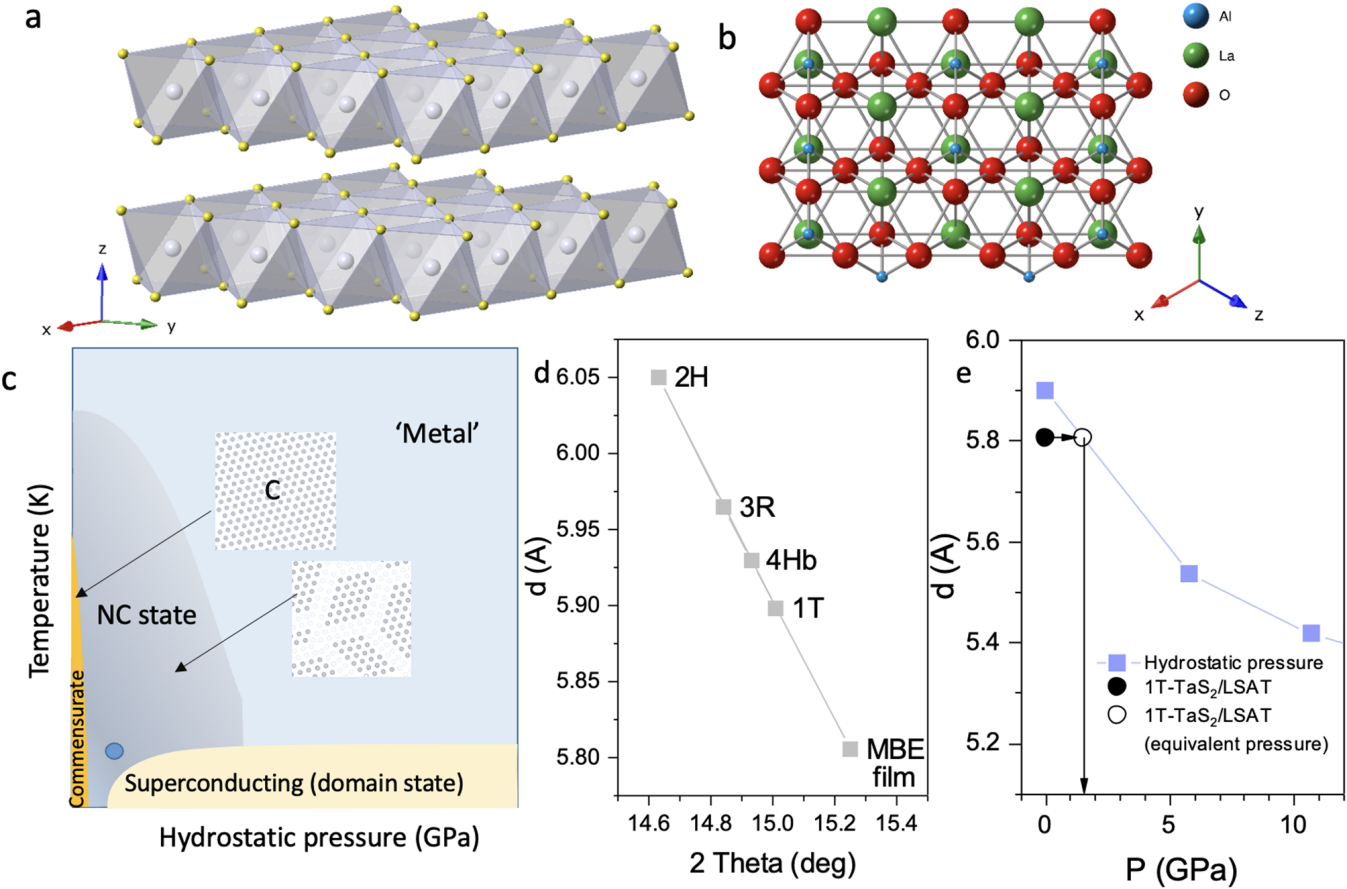
Structure of TaS_2_ and LSAT. **a**) Schematic crystal structure of 1T-TaS_2_. **b**) Schematic LSAT (111) surface structure. **c**) A schematic phase diagram under ambient pressure and hydrostatic pressure. . (adapted from^8^). The blue dot represents this work. **d**) The c-axis inter-layer distances $\:d$ for the different known polytypes of TaS_2_ and the present film on LSAT as a Function of 2$\:\theta\:$ in XRD^35–38^. **e**) The inter-layer distance as a Function of pressure, and the effective pressure of the 1T-TaS_2_/LSAT film (the hydrostatic pressure data are from ref^18^.).

The transition metal dichalcogenides (TMD) exhibit diverse charge ordering phenomena and are of significant current interest both for applications and from the point of view of Fundamental physics and materials science. Amongst them, 1*T*-TaS_2_ (Fig. 1) stands out as a prototype material, which displays a multitude of structural polytypes and charge orders^1,2^. An incommensurate (IC) charge density wave (CDW) is present between $\:\sim550\:$and 350 K, which undergoes a transition to a nearly commensurate (NC) ordered domain state that is present at $\:350-180\:K$, whereupon it undergoes a transition to a commensurate (C) state below ~180 K. This state is particularly interesting because of its unique insulating low-temperature behavior, whose origin has been a long-standing subject of fundamental interest^3–8^. Both the NC and C states have been shown to be useful in device applications^9–15^. 1*T*-TaS_2_ also exhibits a metastable hidden (H) ordered phase, which forms in response to the photoexcitation^16,17^ or to the charge injection through contacts^9^. The latter makes the material very important for potential use in memory devices^11^while the NC state is useful for electronic oscillators, for example^12^. Under hydrostatic pressure, 1*T*-TaS_2_ becomes metallic and superconducting below $\:\sim$5 K^8,18^ (Fig. 1), with the onset of superconductivity at $\:\sim$2.5 GPa. The superconducting phase is also reached by chemical pressure achieved by Se substitution^19^. The growth of thin films of 1*T*-TaS_2_ on common technologically relevant substrates is therefore of significant interest for potential device applications. Thin films may also offer insight into the fundamental physics of the material when the substrate is used to exert an anisotropic lattice strain^20,21^.

So far, 1*T*-TaS_2_ films, ranging from a single layer to a few layers, have been grown by molecular beam epitaxy (MBE) on dichalcogenide substrates^22,23^or on gold (111)^24^and by chemical vapor deposition^25^. Studies of monolayers and few-layer films on substrates^26,27^ showed that their properties are strongly modified with respect to those of the single crystals, or free-standing bilayer films^28^. For example, 1*T*-TaS_2_ monolayers on Au (111) substrates did not show any evidence of CDW formation^24^which occurs in the pristine material^1^. However, a C-CDW is observed on free-standing monolayers^28^. The implication is that the substrate plays an important role in CDW formation, and so the interaction between Au and the 1*T*-TaS_2_ monolayer was indicated to be a possible origin for the modified behavior^24^. Thin exfoliated flakes thicker than $\:10\sim13$ layers were found to revert to the familiar single-crystal properties with the usual CDW phases^26^. At the same time, when $\:40\sim100$ nm thick exfoliated flakes are strained, a strong substrate-induced effect is observed on the transition temperatures between the metastable photoinduced H state and the C state, but less so for the NC-C transition temperature^21^. Such strain effects were previously used to enhance the superconducting critical temperatures in the cuprate superconductors, for example^29,30^, and may be used effectively to tailor the properties of deposited materials. The strains may also be problematic, particularly in devices fabricated by a focused ion beam, and such devices often show very different transport behavior compared to bulk samples^31^.

In this paper, we explore the properties of thin polycrystalline films 1*T*-TaS_2_ grown on LSAT substrates by MBE. Due to the difference in the expansion coefficients, the LSAT substrate exerts a significant in-plane *tensile* strain on the thin film upon cooling, which leads to interesting behavior. We first investigate the film properties using X-ray diffraction (XRD) and atomic force microscopy (AFM). The LSAT/1*T*-TaS_2_ interface is investigated using focused ion beam (FIB)-cut lamellas in combination with high-resolution transmission electron microscopy (HR-TEM). Contrary to unstrained films that show insulating behavior at low temperatures, the transport measurements of 1*T*-TaS_2_/LSAT films show metallic resistivity and the onset of superconductivity with a double transition below $\:T\sim\:3.8$ K. The presence of superconductivity is confirmed by magnetoresistance measurements. We discuss the relevance of the appearance of superconductivity and metallic behavior in the context of the phase diagram of a material on the verge of quazi-2D carrier localization, in which inter-layer interactions play an important role.

## Experimental results

**Fig. 2:**
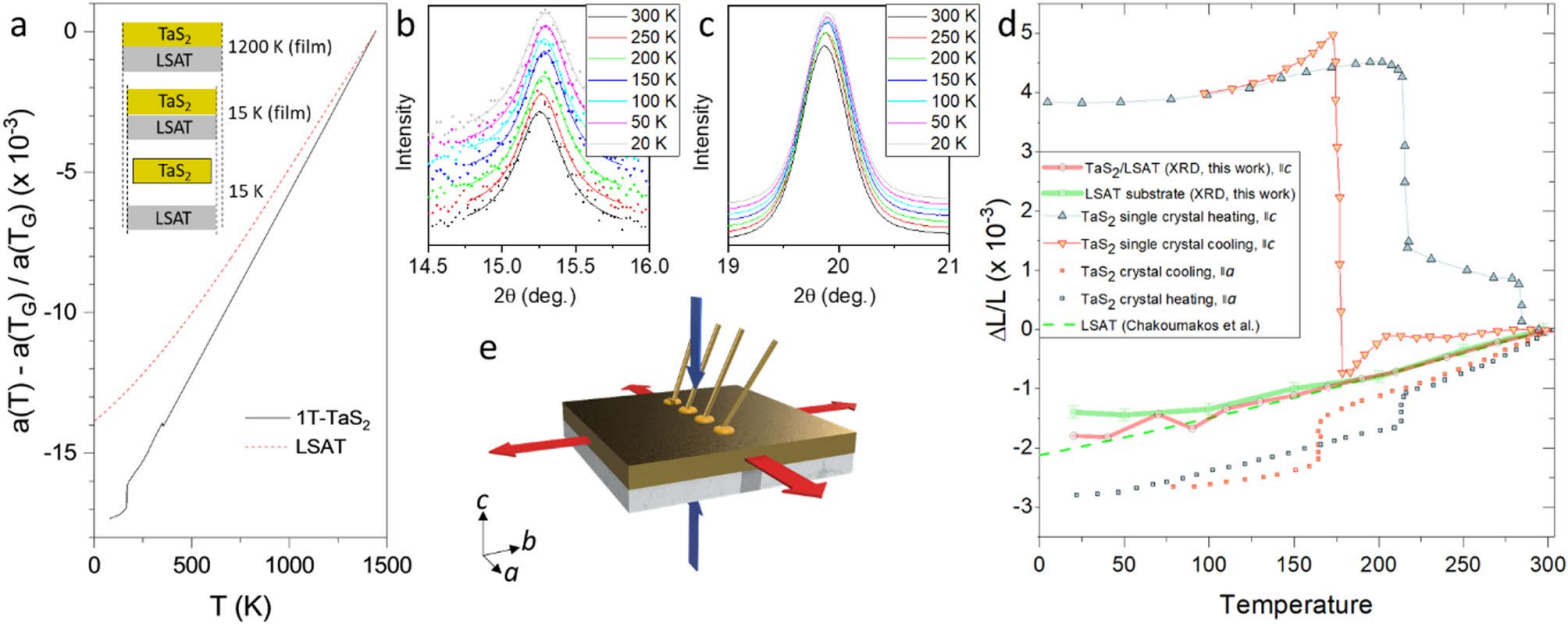
Lattice constants and thin film XRD on TaS_2_/LSAT as a function of temperature. **a**) Differential plot of in-plane lattice constants (//a) for TaS_2_ and LSAT on cooling from the growth temperature T_G_ using data from the literature^32,34^ (The data above 500 K for TaS_2_are extrapolated from refs.^32,33^). The effect of contraction on cooling is shown schematically for the film and for bulk material. **b**) and **c**) Measured XRD data as a function of temperature for TaS_2_ and LSAT respectively. **d**) The differential change in the lattice constants$\:\:\varDelta\:L/L$ (//a and//c) for 1T-TaS_2_/LSAT thin film, 1T-TaS_2_ bulk single crystal and LSAT below room temperature. For the 1T-TaS_2_ crystal, both c-axis and in-plane data are shown, while for TaS_2_/LSAT, only c-axis XRD data are shown. LSAT data from present XRD data (thick red line) are compared with the formula for a-axis thermal expansion from ref^34^. (dashed line). **e**) The schematic drawing shows the direction of the tensile in-plane strain exerted by LSAT on the 1T-TaS_2_ film (red arrows). The accompanying compressive strain along the c-axis is shown by blue arrows. The contacts that are used for resistance measurements are indicated.

The MBE films were grown on (111)-polished LSAT single crystal substrates (Fig. 1), kept at a regulated temperature of 1100 °C, using a Ta evaporator and an S cracker source. (The details of the MBE growth and characterization methods are given in the Methods section.) Different polytypes (2 H, 4Hb and 1 T) grow at different substrate temperatures, and can be easily identified by XRD due to their different inter-layer spacing (Fig. 1d). Here we focus on 1*T*-TaS_2_ grown at T_G_=1100 °C. The thin films are quenched to room temperature by rapidly removing the sample from the heater, following the established procedure for retaining the 1*T*-TaS_2_ polytype, which is stable at high temperatures^1^. During the quenching process performed after growth, a significant differential strain between the film and the LSAT develops, which has an important role in determining the properties of the film at low temperatures. A comparison of the normalized differential in-plane *a* axis lattice expansion of 1*T*-TaS_2_ and LSAT is shown in Fig. 2a using available data from the literature^32–34^ showing that the LSAT contracts less than 1*T*-TaS_2_ on cooling from the growth temperature, thus exerting a tensile strain on the 1*T*-TaS_2_ film.

The XRD data upon cooling for both the TaS_2_ film and the LSAT substrate are shown in Fig. 2b and c. The spot for the XRD measurement was carefully chosen to correspond to the area where resistance contacts were made. The TaS_2_ data (Fig. 2b) do not show any multiple-peak structure, from which we conclude that we are observing a structurally single-phase thin film. The relatively broad diffraction peaks suggest a distribution of the lattice constants, which can be a result of intrinsic strains that leads to relaxation of the structure. The room temperature *c* lattice constant (corresponding to the inter-layer distance), measured for the present film is $\:5.805\pm\:0.02$ Å. This is compared with different polytype interlayer distances for different single-crystal polytype values in Fig. 1d. The value $\:c=5.805$ Å is significantly smaller than the single-crystal value ($\:5.902\left(9\right)$ Å) for the 1*T*-TaS_2_ phase^35^. Such a lattice constant is observed in the 1*T* polytype at a hydrostatic pressure of $\:P\simeq\:\:1.$5$\:\pm\:0.3\:$GPa^18^as shown in Fig. 1e.

The peak at $\:2\theta\:=15.2^\circ\:$, which corresponds to the inter-plane TaS_2_ lattice constant *c*, and the LSAT peak at $\:2\theta\:\simeq\:20^\circ\:$, corresponding to the *a* axis (the crystal is cubic at room temperature) are analyzed by fitting Lorentz line shapes. In Fig. 2d we plot the differential change in the lattice constants $\:{\Delta\:}L/L$ of TaS_2_ and LSAT between room temperature and 10 K, where the temperature dependences of $\:{\Delta\:}L/L$ are compared with in-plane (//*a*) and out-of-plane (//*c*) single crystal data of 1*T*-TaS_2_ from the literature^32,34^. Remarkably, the anomalously large hysteretic *c*-axis expansion associated with the transition to the commensurate state on cooling, which is the hallmark of the 1*T*-TaS_2_ single crystals^32^is absent in the TaS_2_/LSAT thin films. Rather, the *c*-axis shows a monotonic contraction with decreasing temperature, as shown in Fig. 2d, without any indication of a CDW or structural phase transition below room temperature. In contrast, the measured LSAT thermal expansion (Fig. 2d) follows data described in the literature^34^.

**Fig. 3:**
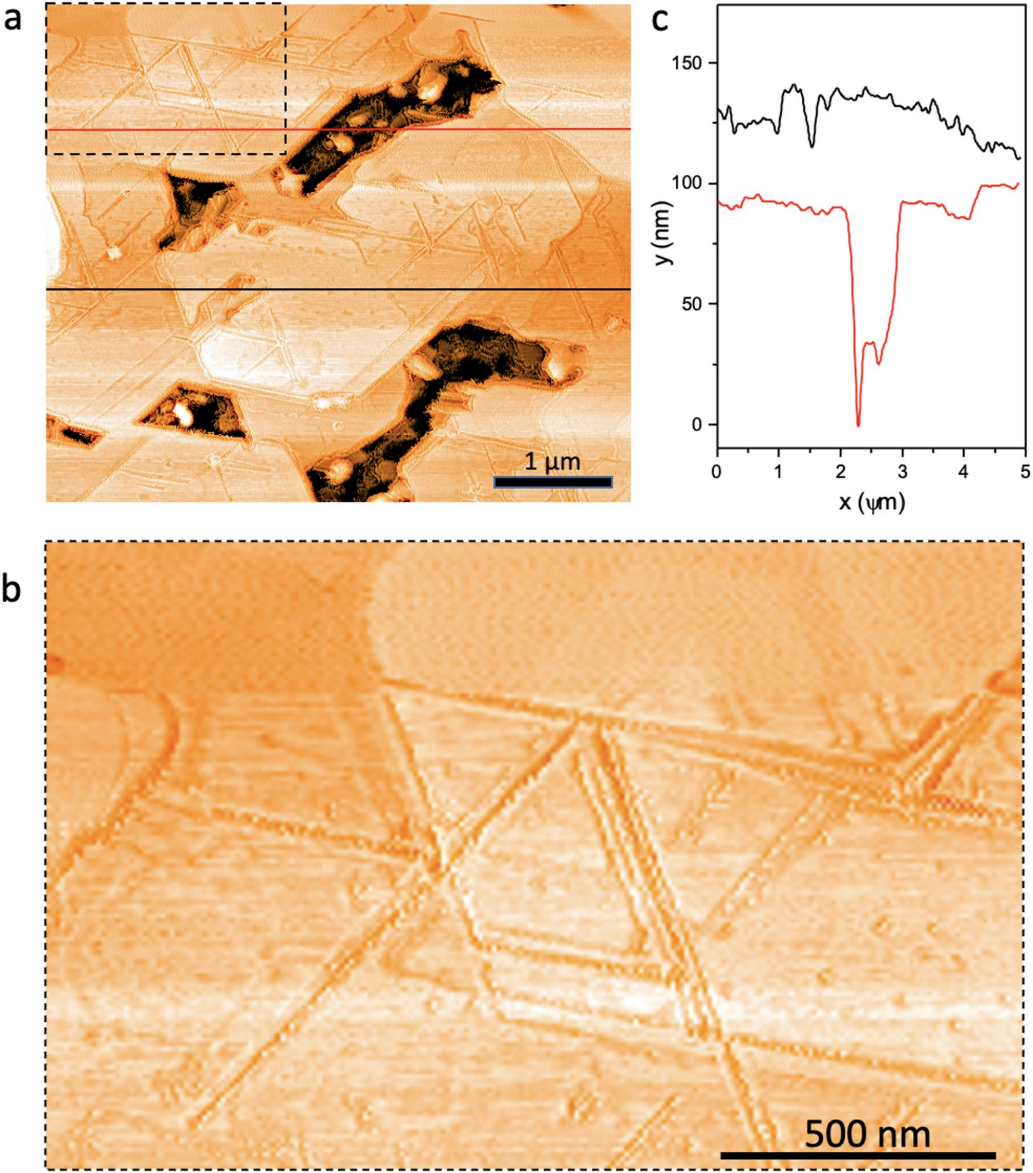
Surface morphology analysis. **a**) An AFM image of the 1T-TaS_2_ film on LSAT. The orientation of the microcrystallites, as determined by their edges, is seen to be the same over the entire image. **b**) A magnified AFM image of the area indicated by the dashed rectangle in panel **a**). **c**) AFM profile scans measured along the red and black lines shown in panel **a**).

AFM measurements (Fig. 3) show uniform coverage of the LSAT substrate by flat-oriented triangular crystallites of TaS_2_. The surface roughness is $\:\sim2\:nm$, shown in Fig. 3c by the measured profile scan measured across the dashed lines indicated in Fig. 3a. The profile scan also reveals the film thickness to be $\:90\pm\:10\:$nm, while the large area flatness over tens of $\:\mu\:$m of $\:\sim10\:nm$. The morphology of the film (shown by the larger magnification AFM image in Fig. 3b) reveals that the islands are oriented, and the edges of the microcrystallites appear to be aligned. The islands are touching each other, which suggests that they are macroscopically connected, which is confirmed by resistance measurements.

**Fig. 4:**
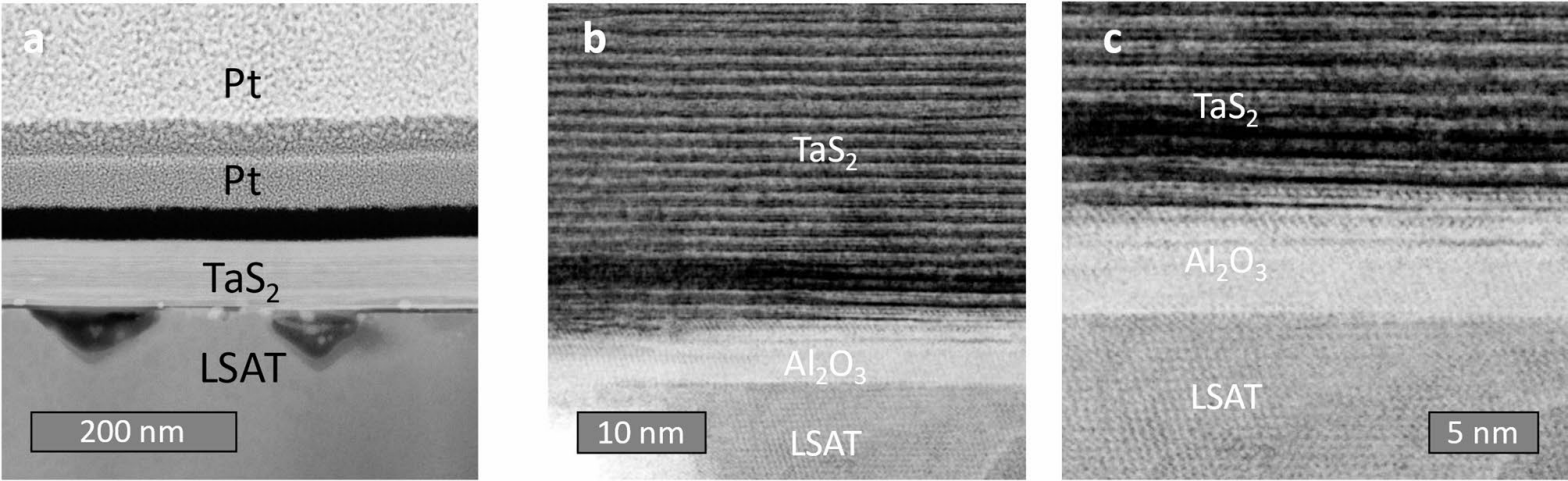
Cross-sectional analysis of a thin film coating. **a**) SEM Image of a lamella showing two protective Pt capping layers deposited with FIB (the thicker bright layer is Pt deposited with an ion beam, and the thinner dark layer is Pt deposited with an electron beam) on top of the sample. **b**,** c**) HR-STEM (HAADF mode) image of a cross-section, clearly showing individual layers in TaS_2_ and the presence of a 6 nm thick Al_2_O_3_ layer between TaS_2_ and LSAT. The chemical composition of the phases present was obtained with EDXS point analysis in a TEM (Fig. 5) as discussed in the methods section. All the images were taken from the same part of a thin film.

**Fig. 5:**
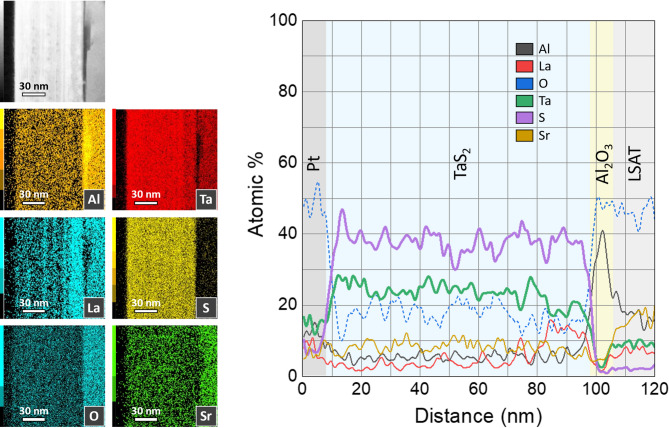
Elemental analysis of the film. **a**) STEM EDXS elemental mapping and **b**) Line-scan chemical analysis of a composition of thin layers of the lamella of the 1T-TaS_2_ sample grown on an LSAT substrate. A 6 nm thick Al_2_O_3_ layer is observed on the LSAT substrate. This layer is formed due to surface degradation of the LSAT because of high-temperature growth, causing the oxidation of the top layers. Intensities of EDXS mapping (**a**) and line-scan (**b**) follow the line profile from the surface of the sample to the LSAT substrate and are the absolute atomic composition of the material. The uniform presence of oxygen is attributed to the post-growth oxidation of the lamella before the EDXS measurement.

The scanning electron microscopy (SEM) and high-resolution scanning transmission electron microscopy (HR-STEM as discussed in the Methods) cross-sectional analyses presented in Fig. 4a clearly show uniform crystalline 1*T*-TaS_2_ layers on top of the LSAT substrate. Figures 4b-c also reveal the existence of a thin buffer Al_2_O_3_ layer between LSAT and the 1*T*-TaS_2_ film. This layer appears during growth and is understood to be a result of a transfer of atoms from the LSAT substrate at the growth temperature. We do not find any substantial areas of an unreacted Ta metal (Fig. 5) in the film or at the interface. In spite of this buffer layer, the 1*T*-TaS_2_ shows fully oriented film growth.

To check whether the buffer layer is solely responsible for the oriented film growth, we have performed a series of growth attempts on pure Al_2_O_3_ substrates. While the substrates have the same composition, we did not manage to obtain TaS_2_ films similar to the ones grown on LSAT. These results imply that the crystalline structure of the films in our work is defined mostly by LSAT rather than the buffer Al_2_O_3_ layer.

Elemental analysis of the cross-section by energy-dispersive X-ray spectroscopy (EDXS) presented in Fig. 5 shows that the buffer layer composition is consistent with Al_2_O_3_ or AlO_x_ (as described in the SI). The 1*T*-TaS_2_ film contains low-level doping of La and Sr, presumed to come from the substrate during the high-temperature growth. Additionally, Pt comes from FIB deposition of the top capping layer (not to scale in Fig. 5). Since the Pt is deposited only in the process of lamella preparation for the EDS measurements, it does not have bearing on the resistivity measurements presented below, where gold paste electrodes were used for making the electrical contacts. The oxygen content measured across the film is attributed to the lamella preparation and handling before the EDS measurement (refer to the SI for additional details).

**Fig. 6:**
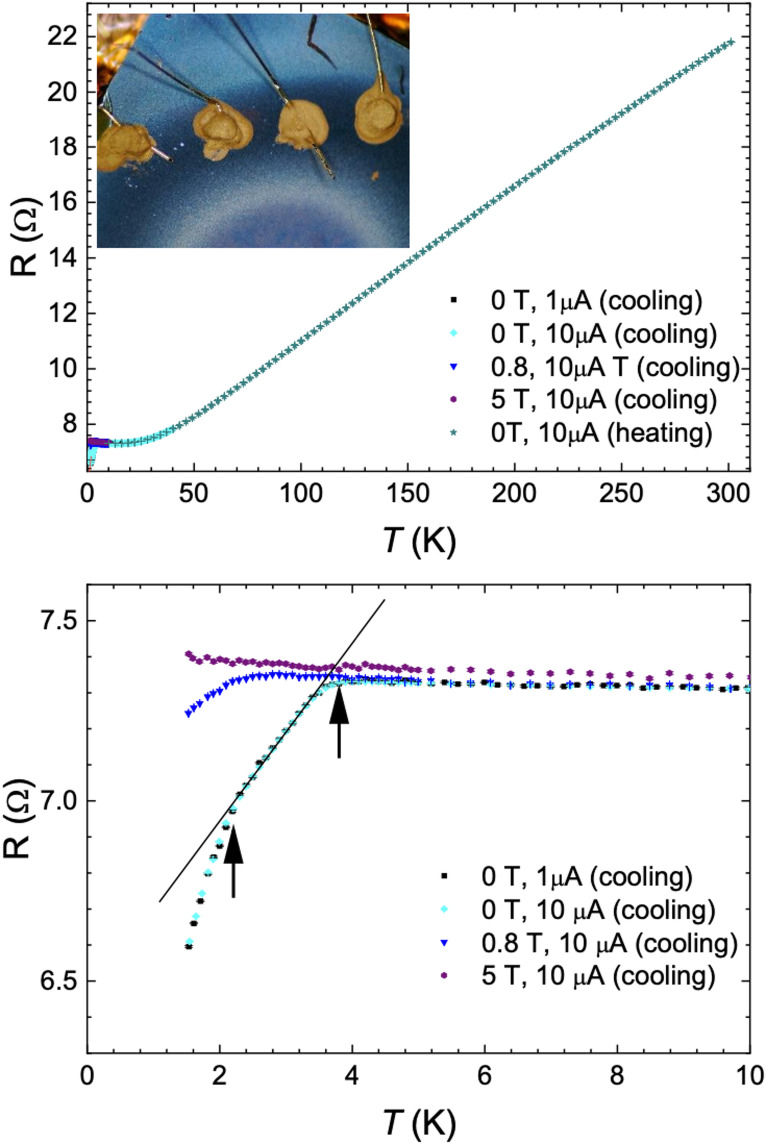
4-probe resistance measurements with and without the external magnetic field. **a**) The resistance as a function of T at B = 0 T, 0.8 T, and 5 T measured on cooling (down) and heating (up). The insert to **a**) shows the contact area on the sample. The resistance slope and T_c_are comparable to the one obtained in ref^8^. under a hydrostatic pressure $\:>8\:$ GPa. **b**) A magnified view of the resistance curve at low temperatures, indicating the presence of two transitions (arrows).

The resistance curves as a Function of temperature, obtained by 4-contact measurements with and without magnetic field are shown in Fig. 6. (The region for placing contacts was chosen based on AFM analysis described above.) For T between 50 K and 300 K, the resistivity is very close to Linear and is not dependent on the external magnetic field. Below 3.8 K, the zero-field R-T curve shows a drop (Fig. 6b). The application of an external magnetic field causes this resistance drop to first diminish, and then disappear (Fig. 6b). The observed behavior is consistent with the onset of superconductivity below 3.8 K, and an upper critical field below 5 T. The films do not show zero resistance down to 1.5 K but appear to show two transitions, the first at 3.8 K and another one near 2.2 K, suggesting that the material is not homogeneous. The observed behavior in the magnetic field unambiguously implies the presence of superconductivity, rather than a phase transition of another kind.

## Discussion

For materials such as 1*T*-TaS_2_, electrons are close to localization^7^, and their properties are strongly dependent on interatomic distances, and particularly, on changes in anisotropy which modify the intra- and inter-planar interactions. To understand the origin of the presently observed behavior, we first analyze the effect of substrate strain. The measured $\:c$-axis lattice spacing is closest to the 1*T* polytype ($\:5.902$ Å), with other polytypes exhibiting significantly larger inter-layer distances (Fig. 1d)^36–38^. The absence of characteristic lattice thermal expansion associated with the commensurate phase of the 1*T* polytype^32^ shown in Fig. 2b implies that there is no transition to the C phase below room temperature.

The indication from the measured room temperature 5.805 Å inter-layer distance is that the 1*T*-TaS_2_/LSAT film has experienced a compressive strain upon cooling from the growth temperature. Such an inter-plane distance ($\:c=5.805\:\text{\AA\:}$) is observed for a sample under a hydrostatic pressure of $\:\sim1.5$ GPa (Fig. 1e). At this value of hydrostatic pressure, the reported superconducting $\:{T}_{c}\:=\:4$ K is close to $\:{T}_{c}\:=\:3.8\:K$ observed here. Assuming that the data on the a-axis thermal expansion from the literature apply to our case, we conclude that the TaS_2_ film experiences a substantial in-plane *tensile* strain along the a axis due to differential contraction of the substrate on cooling, and a compressive strain along the *c*-axis.

It is interesting to compare the measured compressive *c*-axis strain caused by the tensile strain (Fig. 2e), with the (isotropic) strain caused by hydrostatic pressure. Taking a typical value of Young’s (3D) modulus $\:E\simeq\:85$ GPa for 1*T*-TaS_2_, a rough estimate of the compressive strain associated with a hydrostatic pressure of $\:\sigma\:=1.5$ GPa on a bulk sample is $\:{\epsilon}_{c}=\frac{\sigma\:}{E}\simeq\:1.7\times\:{10}^{-2}$. On the other hand, the differential strain $\:{\Delta\:}L/L$ exerted by cooling the TaS_2_ film on the LSAT substrate from 1200 K to 20 K estimated from the thermal expansion data in Fig. 2a is $\:\sim3.5\times\:{10}^{-3}$. Assuming the value of Poisson’s ratio $\:\nu\:=0.26$^39^ for 1*T*-TaS_2_, the calculated *c*-axis *compressive* strain exerted through the conventional Poisson effect is $\:{\epsilon}_{Poisson}=\nu\:\:{\epsilon}\:\sim\:1\times\:{10}^{-3}$. The measured strain $\:\epsilon{}_{c}$obtained from the inter-planar distance is thus more than an order of magnitude larger than expected on the basis of a conventional Poisson effect. The discrepancy may be partly attributed to the inaccuracy of the published values of $\:E$ and $\:\nu\:$^39^, the extrapolation of the lattice constants in Fig. 2a, and the fact that the anisotropy of Young’s modulus was not taken into account by the estimate. Additionally, the spatially inhomogeneous strain exerted by the substrate on the TaS_2_ film may also contribute to the inaccuracy. However, altogether these effects are unlikely to account for the order of magnitude discrepancy between $\:{\epsilon}_{c}$ and $\:{\epsilon}_{Poisson}$. The observation of an anomalously large c-axis compression by XRD in the thin MBE-grown film, in comparison with bulk, implies a non-linear Poisson effect, whereby a relatively small tensile strain by the substrate leads to a large amplification of the *c*-axis compression. We may speculate that the in-plane tensile strain triggers a structural rearrangement that leads to a denser packing of layers and a smaller *c*-axis lattice constant. However, at present we do not have any direct evidence of such a structural rearrangement, and we hope that the work will stimulate further research into the origins of this unusual effect.

The second factor influencing the interlayer distance can be the intercalation of the film with different elements. The elemental analysis of the EDS on our films showed the presence of small amounts of La and Sr (Fig. 5). From the literature it is known, that properties of the metal-intercalated compounds of class A_x_TaS_2_ (where A – metal), including its superconducting transition temperature strongly depend on the amount of intercalated atoms^40,41^. We performed a comparison of grown TaS2 films of different thicknesses and found that only the first few layers of the films are influenced and the amount of intercalates decreases fast with the distance from the substrate.

Previous investigations performed on the metal-intercalated TaS_2_ show a consistent *increase* of the lattice constant *c*, and our results demonstrate a significant *decrease* of *c* when compared to an unstrained crystal. For different intercalated metals (Li, Na, K, Cs etc.) the interlayer distance change varies from 5 to 52% and increases with the metal crystallographic radii^42^. If we consider the parameters for La (ionic radius 1.06 Å for La3+) and Sr (ionic radius is 1.13 Å for Sr + 2), the *c*-axis change can be estimated in a range of 19 to 34% and the inter-plane distance *c* will be $\:6.965-7.843\:\text{\AA\:}$ instead of 5.805 Å. While the amount of intercalates can only be estimated and not calculated precisely, the large Poisson effect shows a significantly stronger impact on the *c*-axis lattice constant when compared to the intercalation.

Irrespective of the discussion above on the mechanism for the amplified compression, the raw XRD data directly shows that the $\:c$-axis inter-layer distance controls the presence of superconductivity and the suppression of the C-CDW phase at low temperatures. The well-documented sensitivity of the electronic structure to inter-layer interactions^5^ and buckling under strain^19^ is consistent with the behavior discussed above. The implication is that relatively small in-plane tensile strains can be used to control macroscopic properties that depend on inter-layer spacing, such as transport. To what extent this applies also to other quasi-2D Van der Waals materials remains to be shown.

Addressing the characteristics of the superconducting phase, the two $\:{T}_{c}$s may indicate the presence of two phases, the lower transition corresponding to $\:P\simeq\:1.5$ GPa, while the 3.8 K transition corresponds to the plateau with $\:{T}_{c}^{P}\simeq\:4.8\pm\:0.5$K observed over a range of hydrostatic pressures $\:1.8\sim20\:$GPa^8,18^. The 1 K difference between the present phase with $\:{T}_{c}=3.8$ K and the high-pressure phase with $\:{T}_{c}^{P}\simeq\:4.8$ K may be attributed to the fact that the present film is strained only in the in-plane direction, not hydrostatically. Finally, we also need to consider the possibility that one of the superconducting phases arises from extrinsic metallic tantalum grains, that may remain after the growth and is not intrinsic to the TaS_2_. The T_c_ of pure Ta metal in the absence of a magnetic field is 4.4820$\:\pm\:$0.0008 K^43^, which is higher than the presently observed $\:{T}_{c}\:$= 3.8 K, suggesting that neither of the observed superconducting phases is due to metallic Ta grains. Moreover, to observe a significant drop in resistance, the amount of Ta would need to be substantial. The HR-STEM images of the FIB cross-sections (Figs. 4 and 5) and AFM scans do not reveal the presence of metallic Ta in quantities that could lead to the observed drop in resistance at 3.8 K.

In addition, the presence of the La intercalation in the grown MBE films can partially explain the presence of the superconducting transition. For the bulk crystals La_0.16_TaS_2_, the observed transition is 2.8 K^41^. In our case, the large Poisson effect has a higher impact on the c-axis lattice constant (causing its decrease instead of an increase), which can also explain the shift of the transition to 2.2 K. Films with thickness < 20 nm show only this phase.

At the same time, it is known that the CDW in a bulk *1T*-TaS_2_ can be suppressed due to the presence of structural defects, and it was shown that an onset to superconductivity can be seen around 2.1 K in the samples with minimal (< 0.1%) presence of Cu atoms^44^. The XRD results in this study displays no change to the *c*-axis lattice constant when compared to a pristine *1T*-TaS_2_ (5.90 Å). Another study on the specially grown *1T*-TaS_2_ single crystals (using CVT method) with a high level of defects (sulphur vacancies) also reports a rapid drop in resistance at 2.5 K and a slight decrease of the c-axis lattice parameter (~ 0.15% from 5.842 Å to 5.833 Å) when compared to the pristine crystals grown by the same authors^45^. While authors do not explicitly discuss the large deviation of their c-axis constant from other literature even in the pure *1T* crystals, it is important to note that the CDW transition temperature which they report in the pure crystals (197 K on cooling) highly differs from the usually reported value (~ 180 K)^9–11^, implying also a deviation in the electronic properties likely related to the structural ones.

In our case, a combination of the small doping with Sr and La (especially in the first 20 nm of the grown films) with the lattice mismatch between the substrate and the film (and the presence of the buffer layer) can be responsible for the formation of the structural defects, which are responsible for the CDW suppression and superconducting transition appearance at 2.2 K.

A possible reason why the resistivity does not reach zero at the lowest temperatures is that the islands are not sufficiently connected, and weak Links prevent the flow of supercurrent. Indeed, zero resistance is not reached down to 1.5 K, implying that the film is not electrically homogeneous. This is similar to the report on the Cu-doped *1T*-TaS_2_, where finite residual resistance is observed down to 0.35 K^44^ and is attributed to “spatially separated regions”. The effect of doping by atoms from the substrate can also result in their accumulation at inter-grain boundaries, hindering the flow of the supercurrent. Inspection of the crystallite boundaries in Fig. 3 suggests that this might be plausible. Another factor influencing the superconducting behavior is non-uniform strain arising from imperfect flatness of the films, whereby the volume fraction of the superconducting phase is small. The metallic shape of the R-T curve with no upturn at low temperatures indicates the absence of the phase-separated insulating islands (commensurate low-temperature C-state) embedded in the metallic film (or vice versa). The Linear R-T curve, with saturation at low temperature is strongly reminiscent of common metals with saturation below 10 K due to the impurity scattering, in accordance with Matthiesen’s rule^46^. We conclude from the broad superconducting transition that the thin films are intrinsically inhomogeneous, but the behavior cannot be easily attributed either to Ta metal inclusions, phase separation or gaps between grains. More likely, inter-grain boundaries, microstrains and possible inhomogeneities in the stoichiometry and impurities may be relevant. A detailed study of the grain topologies may reveal more information to clarify their role in the transport and superconducting properties.

## Conclusions

From the fact that there is no characteristic increase in the resistivity at the NC-C transition or discontinuity in the structural parameters in the region 140–180 K, we conclude that the in-plane lattice strain induced by the LSAT substrate prevents the formation of the commensurate CDW. On the other hand, the in-plane tensile substrate-induced strain appears to have a similar effect as the hydrostatic pressure, compressing the crystal structure along the direction perpendicular to the planes via the Poisson effect, compressing the inter-layer distance, which appears to be beneficial for the appearance of superconductivity. The result is a superconducting phase of 1*T*-TaS_2_ at ambient pressure, that is reached without the need for an elemental substitution or doping with ions.

With this, we have demonstrated the use of Poisson strain engineering for tailoring the superconducting behavior of TMD thin films and an unusual amplification of the Poisson effect resulting from tensile strain exerted by the substrate upon cooling from the MBE growth temperature. The discovery may open the way for various device applications and lead to improving our understanding of the origin of superconductivity in strained charge density wave dichalcogenides.

## Methods

### Thin film growth

*1T*-TaS_2_ films were grown on < 111 > LSAT (5 × 5 × 0.5 mm, one side polished, commercially available from MTI Corporation) substrates using the molecular beam epitaxy (MBE) technique. An ultra-high vacuum system with base pressure of 3e-11 mB, LAB-10 MBE (Scienta Omicron), equipped with EFM3 e-beam evaporator (FOCUS GmbH) and VSS cracker (Dr. Eberl MBE-Komponenten GmbH), was used for sample growth. Substrates were heated to temperatures in the range of 700–1150 °C and exposed to Ta and S beams at small angles to the substrate normal. The deposition rate was set to 0.1 nm/minute and resulted in sample thickness varying from 10 to 100 nm. The resulting films are uniform on a milimeter scale. Departures from uniformity are attributed mainly to the nonuniform temperature across the substrate.

### Samples transfer and handling

The grown films were transferred from the UHV chamber of MBE using a UHV transfer suitcase with a base pressure of 1e-9 mB (Scienta Omicron) or inside an inert argon atmosphere in a sealed container. Between the measurements and manipulations, they were stored in a glovebox with inert nitrogen atmosphere.

### Morphology analysis and polytype identification

Surface morphology analysis was done using a Flex Axiom AFM (Nanosurf) stationed in the glovebox. For polytypes identification, X-ray diffraction (XRD) patterns were obtained with an Empyrean diffractometer (PANalytical). During the measurements, films were kept in a container sealed inside the glovebox to avoid oxidation of the surface.

### Lamella preparation

The (S)TEM samples (lamellas) were prepared using FEI Helios Nanolab 650, FEI, Hillsboro, OR, USA, focused ion beam (FIB). During the sample preparation, samples were protected with a 300 nm thick electron-deposited Pt capping layer and an additional 2.5 μm thick ion-deposited Pt capping layer, which were deposited on top of each other at the selected ion acceleration voltages/beam currents of 20 kV/1.6 nA and 30 kV/0.4 nA, respectively. The sample was extracted with gallium ions at 30 kV/21 nA. In the next step, samples were transferred with an OmniProbe 200 micromanipulator to the EM-tech Cu FIB Lift-out grids where final thinning of lamellas was performed with FIB at 30 kV with step-by-step reduction of beam current from 2.5 nA to 80 pA. Final polishing of lamella was performed with FIB at 1 kV/100 pA for 1 min on each side, enabling the removal of the amorphous residue and gallium artefacts and reaching the desired thickness of < 20 nm, enabling atomic resolution in STEM.

### (S)TEM and elemental analysis

The thin film was analyzed using probe Cs corrected Scanning Transmission Electron Microscope (STEM), Jeol ARM 200 CF, Jeol, Tokyo, Japan, equipped with a high-brightness Cold Field Emission Gun (CFEG) operating at 200 kV. The imaging was performed in high-angle annular dark-field (HAADF) mode to achieve the best resolution for measuring interlayer distance.

Qualitative and quantitative elemental chemical analyses were performed with Energy Dispersive X-ray spectroscopy using Jeol Centurio wide-area Silicon Drift Detector (SDD) system. The positioning imaging for the analysis was done in bright field (BF) mode.

### Resistivity measurements

Resistivity and magnetic field measurements were performed in an Oxford Spectromag cryostat with a superconducting magnet and He exchange gas in the sample chamber to ensure accurate sample temperature measurements. The (in-line) contacts were made with Au paste (Fig. 5).

## Supplementary Information

Below is the link to the electronic supplementary material.

Supplementary Material 1

## Data Availability

The data that support the findings of this study are available from the corresponding author upon request.
