# Supplementary material for: Strain-controlled superconductivity in epitaxially grown thin films of 1T-TaS2
Source: Sci Rep. 2025 Oct 15;15:36052. doi: 10.1038/s41598-025-19901-y (PMC12528373; doi:10.1038/s41598-025-19901-y)
Supplement: Supplementary file 1 — Supplementary Material 1 [file 41598_2025_19901_MOESM1_ESM.pdf]

Supplementary Information

Strain-controlled superconductivity in epitaxially grown thin films of 1*T*-TaS_2

Yelyzaveta Chernolevska[1*], Anže Mraz[1, 2], Rok Venturini[1, 3], Bojan Ambrožič[4], Tomaž Mertelj[1, 4], Goran Dražič[5], Damjan Svetin[1], Damjan Vengust[6], Hsin-Chia Ho[6], Matjaž Spreitzer[6] and Dragan Mihailovic[1, 3, 4]

[1]*Department of Complex Matter, Jozef Stefan Institute, Jamova 39, SI-1000 Ljubljana, Slovenia*

[2]*Faculty for Electrical Engineering, University of Ljubljana, Tržaška 25, SI-1000 Ljubljana, Slovenia*

[3]*Faculty for Mathematics and Physics, University of Ljubljana, Jadranska 19, SI-1000 Ljubljana, Slovenia*

[4]*CENN Nanocenter, Jamova 39, SI-1000-Ljubljana, Slovenia*

[5]*Department of Materials Chemistry, National Institute of Chemistry, SI-1001 Ljubljana, Slovenia*

[6]*Advanced Materials Department, Jozef Stefan Institute, Jamova 39, SI-1000 Ljubljana, Slovenia*

**Corresponding author:* Yelyzaveta.Chernolevska@ijs.si

The oxygen content source in the elemental analysis

One of the important questions is the source of the relatively high oxygen content (15 – 20%) in the grown film, which can be seen in Fig. 5 of the main text. In order to check what exactly the source of this oxygen is, we performed a cross-check of the elemental analysis on a known sample using all the same preparation and experiment steps.

For the check, we took a single crystal *1T*-TaS2 grown via a conventional chemical vapour transport technique with iodine as a transport agent. The structure of the crystal was first confirmed with single-crystal XRD measurement, and its properties were confirmed with standard resistivity measurements on a 50-nm-thick mechanically exfoliated sample. After the resistivity measurements, a lamella was cut directly from the sample as shown in Fig. SI-1.

Depending on the exact position of the measurement, we measured *from 17* up *to 37 atomic %* of oxygen between the crystalline layers of the *1T*-TaS_2 sample prepared this way. Such a high content of oxygen we attribute to the handling of lamellas after their cut and before the EDS measurement. The thickness of a single lamella is ~20 nm (discussed in the experimental methods section of the main text), and its walls are not protected after the cut. This is opposite to the top layer, which is capped by Pt in advance.

The results of a single-crystal oxygen content measurement correspond well to what is obtained on our films (Fig. 5). Thus, we consider the oxide to form during the transportation of the lamellas from FIB to TEM.

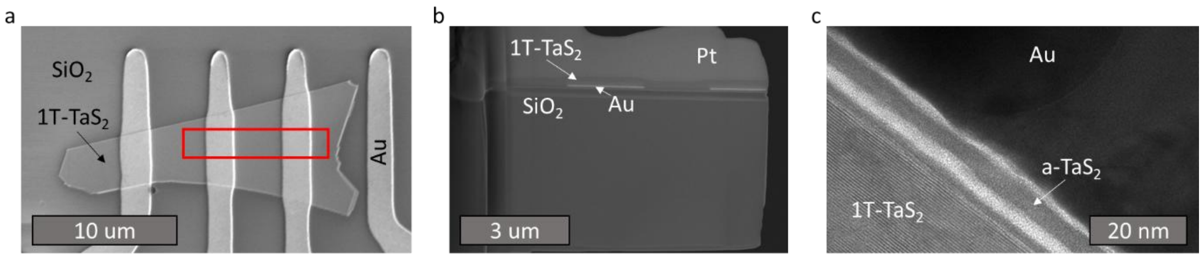

Fig. SI-1. Preparation for elemental analysis on a 50-nm-thick *1T*-TaS_2 flake exfoliated from a single crystal. a) SEM image of the flake before extracting the lamella (zone for the lamella is marked in red). The four Au contacts were used for resistivity measurements. b) Side view of the lamella (SEM) during the extraction. Pt represents the capping layer deposited for sample protection. c) TEM (HAADF) image of the sample where the elemental analysis was performed. a-TaS2 marks the volume where the material was amorphous (refer to the text for details).

Aluminium oxide layer formation and its influence on 1T-TaS_2 growth

<u>Stability of the layer thickness</u>

Cross-sectional analysis of different *1T*-TaS_2 films grown on LSAT substrates shows the presence of a thin buffer layer, composed mostly of Al and O. While we have tried *1T*-TaS_2 growth for different thicknesses between 20 and 100 nm, the thickness of the layer stays constant at around 5-6 nm. The main text discusses results for the 100-nm-thick film, and here (Fig. SI-2), we present elemental analysis and TEM results for a film of ~20 nm. As seen from the figure, a similar 5 nm layer is formed in the case of a thinner *1T*-TaS_2 film.

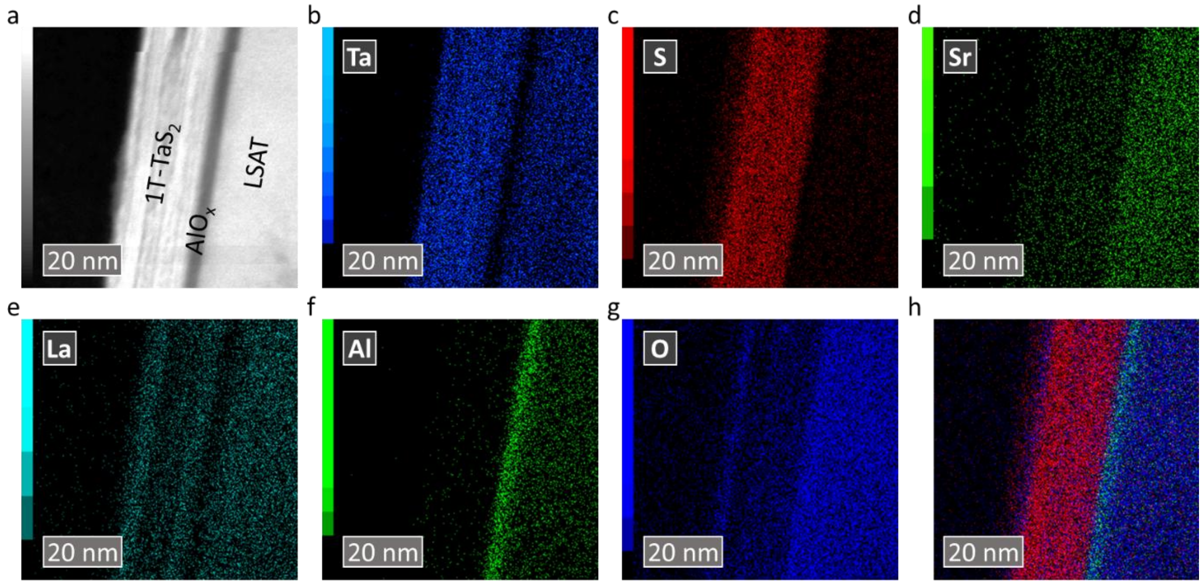

Fig. SI-2. Elemental analysis of a 20-nm-thick *1T*-TaS_2 film grown on LSAT with a buffer AlO_x layer formation. a) TEM image of the film. STEM EDXS elemental mapping for Ta, S, Sr, La, Al and O is presented in panels b-g, respectively. h) A combination of single-element mappings shown in other panels shows a ~5 nm thick layer of AlO_x.

The composition and structure of the buffer layer

While the formation of the buffer layer is, with no doubt, a reproducible and stable process, the exact reason for it is less straightforward. Both elements are present in the substrate material, but while oxygen concentration stays similar in the AlOx layer to what we can observe in LSAT, aluminium concentration increases significantly (Fig. SI-2), implying material transfer from the substrate to its surface.

The exact structure of the layer is not well-known, but from the different experiments performed, we can partially rule out some of the most obvious possibilities.

The performed XRD measurements do not show peaks corresponding to the crystalline Al_2O_3, which can be a result of both the low thickness (and as a result, low signal, which we cannot resolve) and its amorphous structure (AlO_x). The amorphous (or at least, not a conventional Al_2O_3) structure can also be confirmed from our attempts to grow *1T*-TaS_2 directly on Al_2O_3.

To test the nature of the buffer formation and ensure that its XRD signal is not hidden behind the signal of a significantly thicker *1T*-TaS_2 layer above it, we have performed a conditioning experiment, where all the growth parameters were carefully reproduced up to the actual MBE experiment, in the absence of tantalum and sulphur fluxes in the vacuum chamber. This included temperature, time of processing and the substrate manipulation. The resulting substrate was studied in detail using XRD (Fig. SI-3) and compared to the same substrate results before the processing. These studies did not show a significant difference between the two stages of the substrate lifecycle.

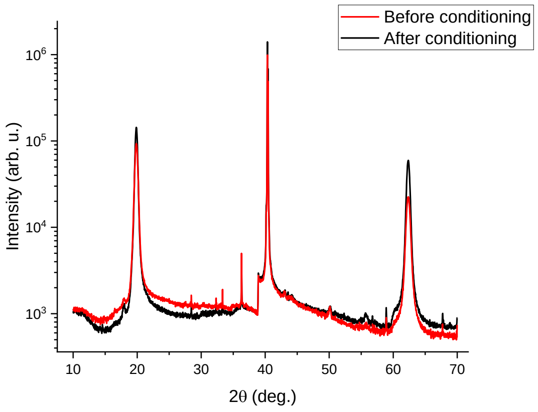

Fig. SI-3. XRD measurement of the LSAT substrate before any thermal processing and after full reproduction of the *1T*-TaS_2 growth parameters with no Ta or S source in the MBE chamber.

Since the composition of the buffer layer formed between our *1T*-TaS_2 films and LSAT substrates resembles Al_2O_3, we tried direct growth on such substrates. For the experiments, we use commercially available substrates with C-plane (0001) and A-plane (11-20) surface orientation. Neither of the attempts resulted in *1T*-TaS_2 films.

From this, we conclude that the properties of the grown films are not defined by a crystalline Al_2O_3 (trigonal, a=4.759 Å) lattice, but probably an amorphous layer. If we consider that the LSAT substrate (cubic, a=3.868 Å) is also not natively compatible with an epitaxial growth for *1T*-TaS_2 (3.36 Å), an amorphous AlOx layer is the only plausible explanation to compensate for the large lattice mismatch.

Temperature dependence of TaS_2 polytypes growth

The different TaS_2 polytypes grow at different temperatures, with *2H* being the lowest temperature and *1T* – the highest temperature structure[1]. From the literature, it follows that the 1T polytype is grown at a higher temperature than 4Hb. This is confirmed by our experiments with the growth done on Si/SiO_2 substrates (Fig. SI-4). The XRD measurements done on the grown films show the peaks corresponding well to the literature data (Fig. SI-4, Fig. 2d).

The formation of *1T* polytype should happen at temperatures above those needed for the *4Hb*. At the same time, in our experiments, when reaching 900 °C, we observe a diffraction peak which is not characteristic of any of the main TaS_2 polytypes (~14.1 deg.)[1,2].

When the same growth parameters are used on the LSAT substrates, we can achieve formation of the *1T* polytype, and the growth improves when the temperature is elevated even higher. Fig. SI-4f shows a comparison of XRD signals for two films grown at 900 and 1000 °C.

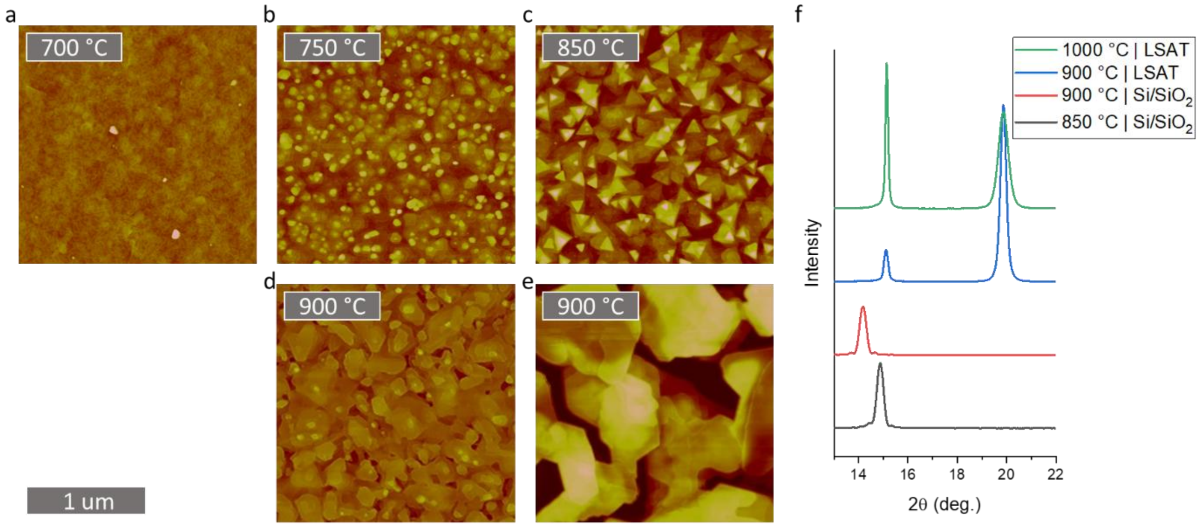

Fig. SI-4. Comparison of TaS2 growth on different substrates. AFM measurements of the films grown at different temperatures on Si/SiO_2 (a-d) and LSAT (e). f) XRD measurement showing *4Hb* signal at ~14.8 deg. (black curve), *1T* signal (~15.2 deg, blue and green curves) and non-identified signal at ~14.1 deg (red curve).

References

1. Di Salvo, F. J., Bagley, B. G., Voorhoeve, J. M. & Waszczak, J. V. Preparation and properties of a new polytype of tantalum disulfide (4Hb-TaS2). *J. Phys. Chem. Solids* **34**, 1357–1362 (1973).
2. Feng, Y. *et al.* 3R TaS2 Surpasses the Corresponding 1T and 2H Phases for the Hydrogen Evolution Reaction. *J. Phys. Chem. C* **122**, 2382–2390 (2018).
